# Non-Protein Coding RNA Genes as the Novel Diagnostic Markers for the Discrimination of *Salmonella* Species Using PCR

**DOI:** 10.1371/journal.pone.0118668

**Published:** 2015-03-16

**Authors:** Ravichantar Nithya, Siti Aminah Ahmed, Chee-Hock Hoe, Subash C. B. Gopinath, Marimuthu Citartan, Suresh V. Chinni, Li Pin Lee, Timofey S. Rozhdestvensky, Thean-Hock Tang

**Affiliations:** 1 Advanced Medical & Dental Institute, Universiti Sains Malaysia, Kepala Batas, Penang, Malaysia; 2 Institute of Nano Electronic Engineering & School of Bioprocess Engineering, Universiti Malaysia Perlis, Kangar, Perlis, Malaysia; 3 Department of Biotechnology, Faculty of Applied Sciences AIMST University, Bedong, Malaysia; 4 Institute of Experimental Pathology, University of Muenster, Muenster, Germany; Naval Research Laboratory, UNITED STATES

## Abstract

Salmonellosis, a communicable disease caused by members of the *Salmonella* species, transmitted to humans through contaminated food or water. It is of paramount importance, to generate accurate detection methods for discriminating the various Salmonella species that cause severe infection in humans, including *S*. Typhi and *S*. Paratyphi A. Here, we formulated a strategy of detection and differentiation of salmonellosis by a multiplex polymerase chain reaction assay using *S*. Typhi non-protein coding RNA (sRNA) genes. With the designed sequences that specifically detect sRNA genes from *S*. Typhi and *S*. Paratyphi A, a detection limit of up to 10 pg was achieved. Moreover, in a stool-seeding experiment with *S*. Typhi and *S*. Paratyphi A, we have attained a respective detection limit of 15 and 1.5 CFU/mL. The designed strategy using sRNA genes shown here is comparatively sensitive and specific, suitable for clinical diagnosis and disease surveillance, and sRNAs represent an excellent molecular target for infectious disease.

## Introduction

Non-protein coding RNAs (npcRNAs or sRNAs) are RNA transcripts capable of performing specific functions but are not translated into protein. sRNAs have been found to play crucial roles in regulating DNA replication, transcription, and mRNA stability [1, 2]. By different experimental strategies, large numbers of sRNA candidates have been identified and shown to be involved in the pathogenesis and cellular regulation [3–7]. Human pathogens including *Helicobacter pylori* [8] and *Vibrio cholerae* [9, 10] have been the subject of immense interest towards the discovery of sRNAs. Previous studies carried out by our group have lead to the discovery of 97 sRNAs from the human pathogen *Salmonella* Typhi, a causative agent of salmonellosis [11].

Salmonellosis a communicable disease caused by *Salmonella* species, remains a constant and non-negligible threat to humans and animals. Exposure to *Salmonella* pathogens is prevalent in a region with poor hygiene/sanitation and improper water treatment. Infection typically occurs through the consumption of *Salmonella*-contaminated food, causing typhoid fever, paratyphoid fever, and non-typhoidal infections [12]. Typhoid/enteric fever is a systemic illness caused by *Salmonella enterica* serovar Typhi (*S*. Typhi) and *S*. *enterica* serovar Paratyphi A (*S*. Paratyphi A). Globally, *S*. Typhi and *S*. Paratyphi A account for more than 21.7 million and 5.4 million infections per year, respectively [12]. These pathogens cause severe health problems and the mortality rate of typhoid fever is 10–30%, mainly among children below 5 years of age [13–16]. Unfortunately, there is no current paratyphoid vaccine, and administration of *S*. Typhi vaccine provides little to no protection against *S*. Paratyphi A [17, 18]. Therefore, there is a fundamental need to develop a rapid diagnostic test for acute clinical management, contact tracing, and identification of convalescent/chronic fecal carriers. Most importantly, detection and differentiation of *S*. Typhi and *S*. Paratyphi A is required for more effective vaccine administration.

Among several proposed diagnostic tests, protein-coding sequence based PCR-amplification is predominantly used, especially for bacterial detections [19]. Sensitivities of 1.8 pg and 1 x 10^3^ of leptospires/mL were achieved via PCR amplification by Ahmed *et al*. [20]. Similarly, Tang *et al* [21] have demonstrated sensitivity and specificity of 93.1% and 89.6%, respectively, using multiplex polymerase chain reaction (mPCR) amplification in detecting *M*. *tuberculosis*. On the other hand, a series of sRNAs were discovered in a number of microorganisms that harbour potential diagnostic markers [10]. Chinni *et al*. have reported the discovery of distinct species-specific sRNAs in S*almonella* [11]. The specificity of these sRNAs for certain *Salmonella* species, including *S*. Typhi and *S*. Paratyphi A, suggest that they might be a potential target for diagnostics. In the present study, we have designed suitable sequences to selectively amplify the sRNAs by mPCR for the detection and differentiation of salmonellosis to be used as molecular markers for the diagnosis of infectious disease.

## Materials and Methods

### Bacterial isolates and genomic DNA extraction

Twenty-two *Salmonella* species and 15 other bacterial strains (Gram-negative and Gram-positive) were obtained from both the Veterinary Research Institute, Ipoh, Malaysia and Advanced Medical and Dental Institute, Universiti Sains Malaysia. Genomic DNA extraction was performed using an in-house protocol. In brief, the bacterial strains were cultured in Luria–Bertani (LB) broth for 16 h (37°C, 200 rpm). Subsequently, 1.5 mL of each culture was centrifuged (13,000 rpm, 1 min), and the supernatants were discarded. The pellets were resuspended in 200 μL of Solution I (20% sucrose, 50 mM Tris-HCl [pH 8.0], 1% SDS, 0.2 M NaOH, 25 mM EDTA [pH 8.0], and 0.1 M NaCl), which was followed by addition of 200 μL of Solution II (3 M sodium acetate, pH 6.4). The tubes were gently inverted, incubated for 5 min at room temperature, and centrifuged (13,000 rpm, 5 min). The supernatants containing DNA were then transferred to fresh tubes. One mL of 100% ethanol was added to each tube, followed by 40 μL of 3 M sodium acetate (pH 5.2) to precipitate the genomic DNA. The tubes were then incubated at—80°C for 20 min and centrifuged (13,000 rpm at 4°C, 13 min). The DNA pellets were washed with 1 mL of 70% ethanol, air-dried, and dissolved in 50 μL sterile distilled water.

### Strategy for PCR-based diagnostic targeting of sRNA genes for the detection and differentiation of salmonellosis

The mPCR was designed to amplify three sRNA genes (*StyR-3*, *StyR-36*, *StyR-143)* and a control plasmid DNA. *StyR-3* is present in all *Salmonella* species. *StyR-36* is *S*. Typhi specific, whereas *StyR-143* are shared by *S*. Typhi and *S*. Paratyphi A. Thus, in cases of *S*. Typhi infection, *StyR-3*, *StyR-36* and *StyR-143* should be amplified, whereas only *StyR-3* and *StyR-143* should be detected in *S*. Paratyphi A-infected samples. To rule out false negative results due to the presence of PCR inhibitors, an internal amplification control (IAC) plasmid (p*L50*) that yields a 650bp product was included in the reactions.

### mPCR amplification and PCR product analysis

PCR amplification was performed in a 20 μL reaction volume, which contained the following: 0.25 μM each forward and reverse primer for *StyR-3*, *StyR-36*, *StyR-143*, as well as *pL50* (Bio Basic Inc., Toronto, Canada); 200 μM dNTPs; 3 mM MgCl_2_; 2 U Taq DNA polymerase (Biotools, Spain) in 1X PCR buffer (10 mM Tris-HCl, [pH 8.3], 50 mM KCl). For template DNA, we used 1 μL (100 ng) of genomic DNA extracted from bacterial cultures or 2 μL of DNA extracted from spiked stool samples. PCR was performed in a Bio-RAD (USA) thermocycler with an initial denaturation at 95°C for 1 min, followed by 30 amplification cycles (30 s denaturation at 95°C, 30 s annealing at 66°C, 30 s extension at 72C), and a final elongation of 2 min at 72°C. The PCR products were analyzed by electrophoresis using a 2% agarose gel in TAE buffer (40 mM Tris acetate, 1 mM EDTA [pH 8.0]) containing 0.5 μg/mL of ethidium bromide. The PCR reaction products (20 μL) were electrophoresed at 60 V for 60 min and visualized using a gel-imaging system (Bio-RAD, USA).

### Optimization of the mPCR

Optimization of the mPCR was carried out to maintain a balanced amplification of all the targeted regions of the genomic DNA. To begin with, a range of annealing temperature from 60 to 70°C was used for the amplification. To optimize the concentration of MgCl_2_, different concentrations used were within the range from 0.5 to 4.5 mM, in increments of 0.5 mM. Taq Polymerase optimization was also carried out, whereby different amounts of 1.0, 1.5, 2.0 and 2.5 units were employed for amplification.

### Sensitivity and specificity of the mPCR assay

We used 37 different bacterial strains to assess the analytical specificity of the optimized mPCR assay. The analytical sensitivity represents the lowest concentration of template DNA that can be amplified to produce visible bands upon gel electrophoresis. To determine assay sensitivity, genomic DNA from *S*. Typhi, *S*. Paratyphi A, and *S*. Paratyphi B (i.e., non-*S*. Typhi *and* non-*S*. Paratyphi A) was extracted and serially diluted 10-fold. An artificial stool contamination experiment (stool spiking) was also carried out based on a method described by Kongmoung *et al*. [22], with slight modification. Single colonies of *S*. Typhi and *S*. Paratyphi A were inoculated and grown overnight in LB broth. The bacterial cultures were adjusted to 0.5 of MacFarland turbidity standard, which is equivalent to 1.5 x 10^8^ CFU/mL. A ten-fold serial dilution was performed, and 1 mL of each dilution was spiked into 0.2 g of healthy human stool (confirmed to be *Salmonella* negative through culturing). The infected feces were then mixed with 9 mL of selenite cysteine broth. The spiked stool samples (before and after enrichment) were processed for PCR amplification. In brief, 1.5 mL of each spiked stool sample was centrifuged (13,000 rpm, 1 min). The supernatants were then discarded, and the pellets were washed with 500 μL of 0.01 M phosphate-buffered saline (PBS). Next, 50 μL of chelex slurry (10% 200–400 mesh, Bio-RAD, USA) was added and boiled for 10 min. Following centrifugation (13,000 rpm, 15 min), 2 μL of each of the supernatants was used as template in the mPCR assay.

## Results and Discussion

Small non-protein coding RNAs are ~18–500 nts long, untranslated RNAs that participate in various cellular processes, from housekeeping to virulence and pathogenesis. Moreover, some of these molecules have been suggested as molecular markers of genetic diseases and cancer [23–30]. Although growing evidence has indicated that sRNAs can serve as biological markers for human diseases, investigation into the use of small sRNAs as targets for the diagnosis of infectious agents has not been explored exhaustively.

Previous PCR-based assays for diagnosing salmonellosis chiefly have been based on the detection of protein-coding genes or 16S rRNA genes [31–35]. In the present study, we have demonstrated the efficacy of using sRNA genes as molecular targets for detecting and differentiating *S*. Typhi and *S*. Paratyphi A. Specifically, we have tested three previously identified sRNA candidates as potential markers for *Salmonella* infection. Notably, these sRNAs have been reported to be specific for all-*Salmonella* species, *S*. Typhi only, or both *S*. Typhi *and S*. Paratyphi A. Taking advantage of the differing specificities of these sRNA gene candidates, we were able to detect salmonellosis and further differentiate *S*. Typhi *and S*. Paratyphi A by mPCR assay.

### Selection of the candidate sRNA diagnostic markers and development of the mPCR assay

**Fig 1:**
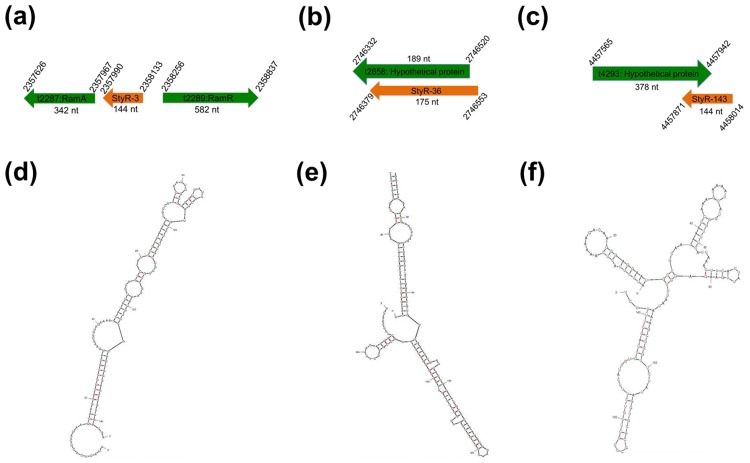
Genomic location and predicted secondary structures of non-protein coding RNAs. (a-c) Schematic representations of *S*. *typhi* sRNA genes. Coordinates of depicted genes are based on the completed genome of *S*. *typhi* Ty2 (AE014613). Drawings are not according to scale. (d-f) Predicted secondary structures of StyR-3, StyR-36 and StyR-143 sRNAs, respectively using mfold programme.

To investigate the efficacy of using sRNA genes as molecular markers for salmonellosis, three sRNA genes (*StyR-3*, *StyR-36*, *StyR-143)* were selected for mPCR development (Table 1). *StyR-3* (GenBank accession no: FJ746361.1) [11] is a promoter-associated sRNA gene that is co-transcribed with the *ramA* gene (mediates multidrug resistance) and overlaps with the DNA binding site of the RamR repressor (Fig. 1a). The 144-nt *StyR-3* RNA was shown to be present in all *Salmonella* species via bio-computational analysis. On the other hand, *StyR-36* (175 nt) is present only in *S*. Typhi and located between nucleotides 2746553 and 2746379 overlapping the 5’-UTR of hypothetical protein-coding *t2658* gene (*S*. *typhi* Ty2 genome GenBank accession no: AE014613) [36] (Fig. 1b). Therefore, detection of *StyR-36* in our mPCR assay specifically indicates *S*. Typhi infection. The third biomarker candidate analyzed in this study was *StyR-143* (144 nt) (GenBank accession no: FJ746389.1) [11], which is present in both *S*. Typhi and *S*. Paratyphi A. *StyR-143* is antisense to the 3'-end of the open reading frame (ORF) of the hypothetical protein-coding *t4293* gene (Fig. 1c). Using mfold programme, the secondary structures of the sRNAs were predicted (1d-f).

**Table 1:** *StyR-3*, *StyR-36* and *StyR-143* sRNA gene sequences.

sRNA gene candidates	Sequence (5’–3’)
*StyR-3*	TTACTCACTCATAATCAAGGGCTGCCGCATGAAGTGGTAGAAAAGCATATTGCAGGCCATGCGATAAGCCGTCTCACAATTTGTGTGGTTATTACTATGCTTATTGCTGTTGCCGTAAATGTGCGGTGCGGGAGCCGCTGACGA
*StyR-36*	CCATGCGCTTGCGCTAAGAGACGTCAGGTATCTATGGAGGAACAAGTTATGGATACAAACGAACTTGGCTTAGTTAAGGCGCGTGTTGAACTGATCACCGCTATGCTCAAATGCGCAACCGCGTTTGTTGGCTTAGTTGGTGCGGTTTACGCCGTTCTTAACATGGCCTTCAACT
*StyR-143*	ATTCTTACTCAAAAAGACAAGGGAGGAATGCCGCAAGAAACCAAAGAAAATCATGGGTTTCATTAAACTTCATTATTGAAGAGGTTTAATAAAGCTGGTTCTATAGGTGCGCGCCTGCTCGTCTTTCATTGTGCCAGCTTTTCT

Subsequent to this, the analyses of GC content of these sRNA genes were also computed. The percentage of GC content is 47.91, 39.58 and 48.58% for *StyR-3*, *StyR-143* and *StyR-36*, respectively. This implies the high efficiency of PCR amplification associated with these genes, as the PCR amplification efficiency increases with lower GC content (less than 50%). PCR amplification of the 475bp product that corresponds to the *StyR-3* gene indicates the presence of *Salmonella* species within the sample, whereas detection of the *StyR-143* band (304bp) demonstrates the presence of *S*. Typhi and/or *S*. Paratyphi A. *S*. Typhi can be specifically identified through a 204bp product, which is amplified from the *S*. Typhi-specific *StyR-36*. Our strategy for differentiating *S*. Typhi and *S*. Paratyphi A is based on the fact that the *StyR-3*, *StyR-143* and *StyR-36* bands should be detected for *S*. Typhi-infected samples (475bp, 304bp and 204bp bands, respectively). However, in the case of *S*. Paratyphi A infection, only the 475bp (*StyR-3*) and 304bp bands (*StyR-143*) should be observed.

**Fig 2:**
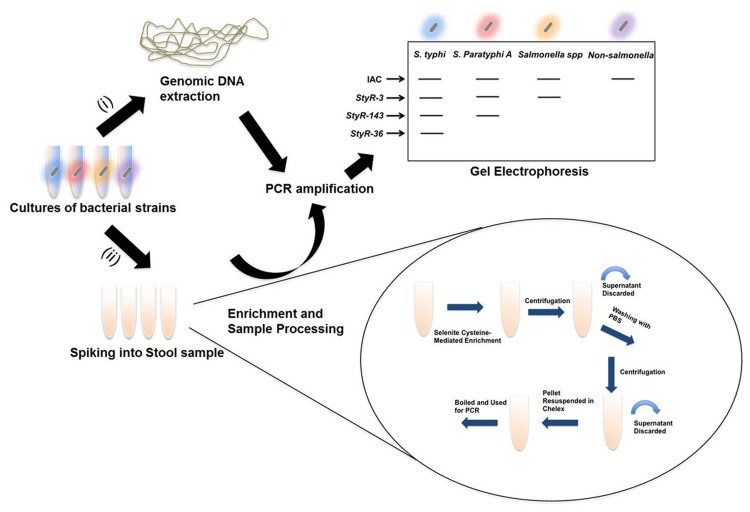
Summarized strategy of mPCR. Overnight cultures of the bacterial strains were subjected to (i) genomic DNA extraction to determine specificity and sensitivity of the mPCR assay (ii) determination of detection limit in spiked fecal samples. Following genomic DNA extraction, PCR amplification was carried out using primers designed based on pL50 (IAC), *StyR-3*, *StyR-143* and *StyR-36* genes and analyzed by gel electrophoresis.

Furthermore, to rule out false negative results due to the presence of PCR inhibitors, an IAC plasmid was incorporated into the PCR to yield a 650bp product (Table 2). PCR inhibitors (such as bilirubin and bile salts) are known to be present in fecal samples. For this reason, we utilized a 10% chelex solution during genomic DNA extraction. Chelex prevents DNA degradation and removes PCR inhibitors from samples to avoid false negative results [37]. Fig. 2 summarized the PCR strategy used in this study.

**Table 2:** Primers used in the mPCR.

Target gene	Primer nameSequence (5’–3’)	Target serovars	Amplicon size (bp)
*StyR-3*	StyR-3 F ACCTTTGAAAAGTACCTTGACGGCGTAC StyR-3 R GCTGCGAATCAAAACCATACTTGAGACC	*Salmonella* genus	475
*StyR-36*	StyR-36 F TGCCATGTAATCGGACGCCGAC StyR-36 R AGCCAACAAACGCGGTTGCG	*Salmonella* Typhi	204
*StyR-143*	StyR-143 F CGCTCCTCCACATCCAACAGTGAG StyR-143 R ATGAACAAGTGGAAACCTGGCACG	*Salmonella* Typhi and *Salmonella* Paratyphi A	304
Plasmid pL50	L50F GTCTACCAGGCATTCGCTTCAT L50R CTGTGAATGCTGCGACTACGAT	Internal Amplification Control	650

### Standardization of mPCR

**Fig 3:**
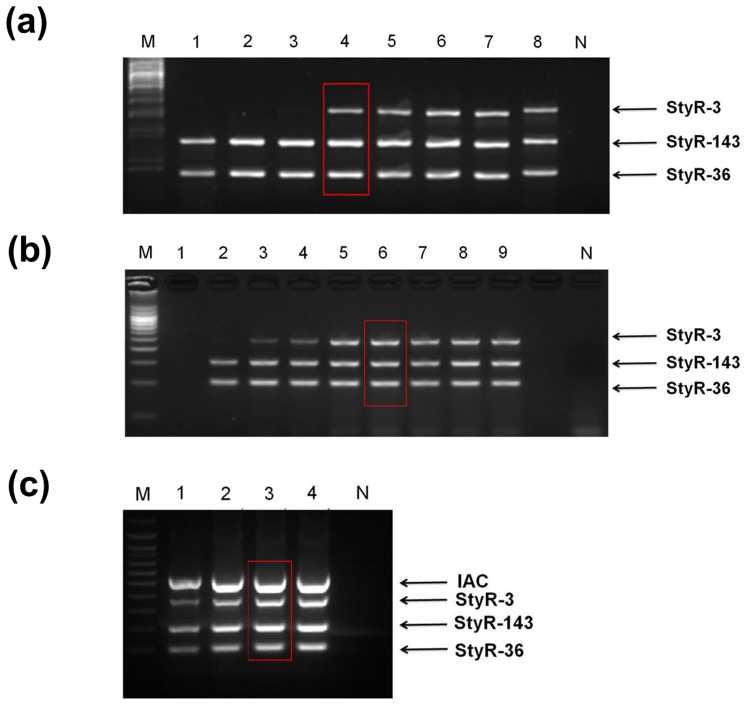
Standardization of multiplex PCR. **(a) Optimization of annealing temperature.** Lane M: 100 bp DNA ladder (Promega), Lane 1: 70°C, lane 2: 69.2°C, lane 3: 68°C, lane 4: 66°C, lane 5: 63.7°C, lane 6: 61.9°C, lane 7: 60.7°C, lane 8: 60.0°C, lane N: Negative control **(b) Optimization of MgCl**_**2**_ **concentration.** Lane M: 100 bp DNA ladder (Promega), Lane 1: 0.5 mM, lane 2: 1.0 mM, lane 3: 1.5 mM, lane 4: 2.0 mM, lane 5: 2.5 mM, lane 6: 3.0 mM, lane 7: 3.5 mM, lane 8: 4.0 mM, lane 9: 4.5 mM, lane N: Negative control **(c) Optimization of the amount of Taq polymerase.** Lane M: 100 bp DNA ladder (Promega), Lane 1: 1.0 unit, lane 2: 1.5 unit, lane 3: 2.0 unit, lane 4: 2.5 unit, lane N: negative control.

mPCR consists of one or more different amplicon systems combined in a single run. Hence, the parameters chosen must be optimal to ensure an even amplification efficiency for all the systems involved. An important criterion in developing mPCR is annealing temperature optimization. In this study, the mPCR developed involves the annealing of three different sets of primers against the genomic DNA template. The result showed that the optimum annealing temperature for amplification of all primers was 66°C, which is the highest temperature of the range from 60 to 66°C (lane 4, Fig. 3a). Compared to uniplex PCR analysis, mPCR is associated with higher concentration of MgCl_2_, as more primers used requires even more extensive neutralization action of the phosphate group's negative charges by Mg^2+^ ions. In this study, optimization of PCR was carried out with different concentrations of magnesium chloride from 0.5mM to 4.5mM. An even amplification was observed from 2.5mM to 4.5mM (lane 5–9, Fig. 3b) while concentrations lower than 2.5mM showed an uneven amplification of the targets (lane 1–4, Fig. 3b). The optimal MgCl_2_ concentration for this mPCR was determined to be 3mM (lane 6, Fig. 3b). This value was chosen over the other values as too much of free Mg^2+^ may increase nonspecific products. Although this concentration produced similar amplification efficiency compared to 2.5mM, 3.0mM was chosen, taking into consideration of the incorporation of IAC in the future. This value is also in concordance with several studies that involves mPCR [38–41]. Subsequent to this, optimization of *Taq* polymerase was carried out with the inclusion of plasmid DNA (100ng of pL50 plasmid). The amounts of *Taq* polymerase driving the PCR ranged from 1.0 to 2.5 U (lane 1–4, Fig. 3c). A visible and clear amplification of all targets including the IAC was observed with 2.0 U and 2.5 U of *Taq* polymerase (lane 3–4, Fig. 3c). The results demonstrated that 2.0 U of *Taq* polymerase was the optimal concentration for amplification (lane 3, Fig. 3c). This is corroborated by the finding that a high PCR efficiency is achieved when concentration of *Taq* polymerase falls within the range of 2 U in a 25 μL of PCR reaction volume [39] (lane 3, Fig. 3c).

### Determination of genomic DNA detection limit

**Fig 4:**
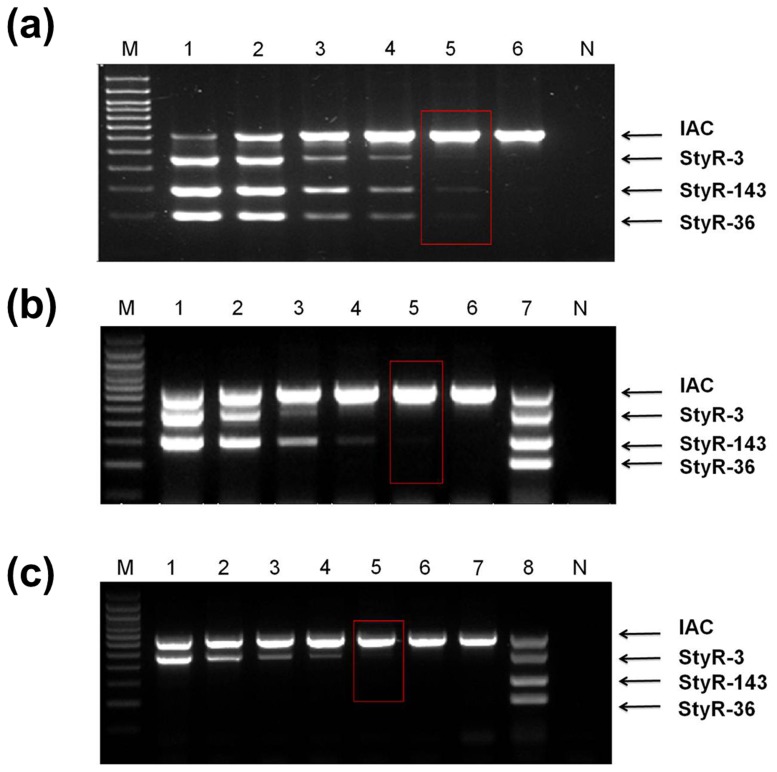
Analytical sensitivity of serially diluted genomic DNA on 2% agarose gel electrophoresis. **a) *S*. Typhi.** Lane M: 100 bp DNA ladder (Promega), Lane 1–6: Serially diluted gDNA from 100 ng to 1 pg, Lane N: negative control **b) *S*. Paratyphi A.** Lane M: 100 bp DNA ladder (Promega), Lane 1–6: Serially diluted gDNA from 100 ng to 1 pg, Lane 7: *S*. Typhi gDNA, Lane N: negative control and **c) *S*. Paratyphi B.** Lane M: 100 bp DNA ladder (Promega), Lane 1–7: Serially diluted gDNA from 100 ng to 100 fg, Lane 8: *S*. Typhi gDNA, Lane N: negative control.

The mPCR assay developed involves three different regions of genomic DNA (gDNA), namely the loci of StyR-3, StyR-143 and StyR-36 RNAs, targeted by three different primer pairs. The limit of detection was determined for each of these different regions of gDNA. In order to determine the detection limit, mPCR was performed using different concentrations of template genomic DNA (*S*. Typhi, *S*. Paratyphi A and *S*. Paratyphi B) serially diluted ranging from 100ng to 1pg. The results indicated that the limit of detection of the optimized mPCR test is about 10pg of template DNA (*S*. Typhi, *S*. Paratyphi A *and S*. Paratyphi B) (lane 5, Fig. 4a-c). This is equivalent to approximately 10^3^ bacteria (assuming that one bacterium contains 3.48 fg of genomic DNA) [42,43] and is in accordance with previous studies [44–47].

### Determination of mPCR assay specificity and sensitivity

**Fig 5:**
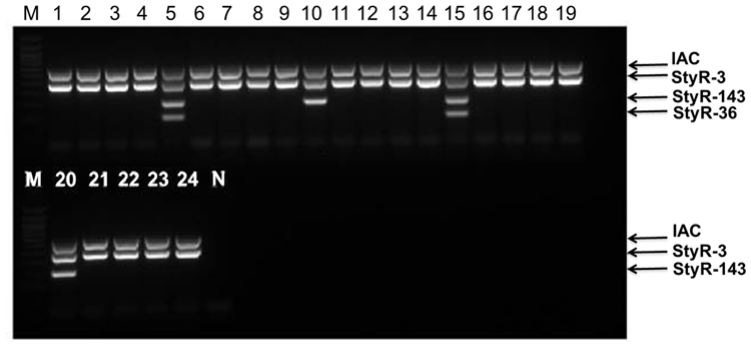
Representative agarose gel of amplified mPCR products using genomic DNA from Salmonella strains. Lane M: 100bp ladder (Promega), lane N: Negative control, lane 1: *S*. Pullorum, lane 2: *S*. Jawa, lane 3: *S*. Kedougou, lane 4: *S*. Mikawashima, lane 5: *S*. Typhi, lane 6: *S*. Give, lane 7: *S*. Hadar, lane 8: *S*. Corvallis, lane 9: *S*. Rissen, lane 10: *S*. Paratyphi A, lane 11: *S*. Bareilly, lane 12: *S*. Newport, lane 13: *S*. Agona, lane 14: *S*. Tennessee, lane 15: *S*. Typhi, lane 16: *S*. Typhimuruim, lane 17: *S*. Weltevreden, lane 18: *S*. Enteritidis, lane 19: *S*. Albany, lane 20: *S*. Paratyphi A, lane 21: *S*. Paratyphi B, lane 22: *S*. Paratyphi C, lane 23: *S*. Braenderup, lane 24: *S*. Infantis.

**Fig 6:**
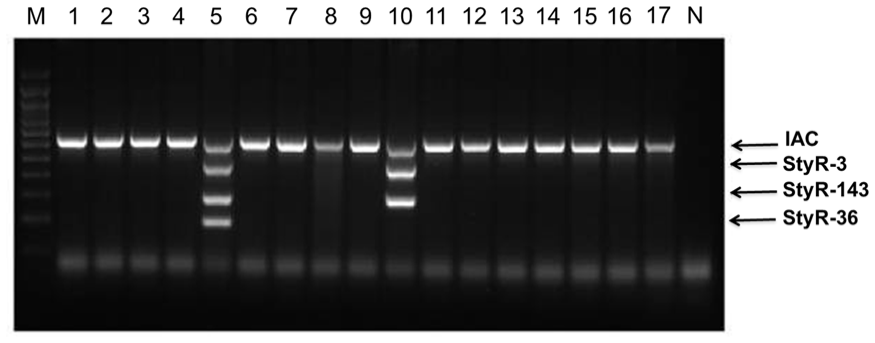
Representative agarose gel of amplified mPCR product using genomic DNA from non-salmonella species. Lane M: 100bp ladder (Promega), lane N: Negative control, lane 1: *Klebsiella pneumonia;* lane 2: *Pseudomonas aeruginosa*, lane 3: *Escherichia coli*, lane 4: *Shigella flexnari*, lane 5: *Salmonella* Typhi, lane 6: *Vibrio cholerae*, lane 7: *Acenitobacter baumannii*, lane 8: *Aeromonas hydrophila*, lane 9: *Neisseria meningitidis*, lane 10: *Salmonella* Paratyphi A, lane 11: *Streptococcus spp*, lane 12: *Staphylococcus epidermidis*, lane 13: *Providencia spp*, lane 14: *Enterococcus feacalis*, lane 15: *Citrobacter freundii*, lane 16: *Proteus mirabilis*, lane 17: *Serratia marcerscens*.

We tested the specificity of our mPCR assay by examining a total of 37 bacterial strains (Table 3). We found that the mPCR assay successfully amplified DNA from all of the *Salmonella* strains tested (lanes 1–24, Fig. 5), which was detected through the *Salmonella* species-specific *StyR-3* amplicon (475bp). Furthermore, *S*. Typhi infections were specifically detected through amplification of the 204-bp product from the *StyR-36* gene (lanes 5 and 15, Fig. 5). The *StyR-143* PCR product (304bp) was present in both *S*. Typhi- *and S*. Paratyphi A-positive samples. Therefore, amplification of the *StyR-143* gene indicates infection with *S*. Typhi and/or *S*. Paratyphi A (lanes 5, 10, 15, and 20; Fig. 5). For this reason, amplification of both *StyR-36* and *StyR-143* can distinguish *S*. Typhi- and *S*. Paratyphi A-infected samples. A common laboratory test often misses the detection of S. Paratyphi A as this microorganism does not produce hydrogen sulfide. Hence, specific detection of S.Paratyphi A by PCR based on the specificity of StyR-143 is important for the accurate diagnosis of paratyphoid fever. This PCR-based test can also obviate the usage of the Widal test that is dependent on the production of antibodies over a period of 2–3 weeks. Fifteen non-Salmonella species tested showed no amplification for StyR-3, StyR-36 and StyR-143, with only the IAC detected, indicating an optimal degree of specificity for our assay (Fig. 6). Repeated PCR testing of the sensitivity and specificity of the primers revealed similar reproducible results.

**Table 3:** Bacterial samples used for the evaluation of primer specificity in the mPCR assay and the results.

	sRNA genes		
Bacterial sample	*StyR-3*	*StyR-143*	*StyR-36*
***Salmonella enterica* Serovar**			
Pullorom	**+**	**-**	**-**
Jawa	**+**	**-**	**-**
Kedougou	**+**	**-**	**-**
Mikawashima	**+**	**-**	**-**
Typhi	**+**	**+**	**+**
Give	**+**	**-**	**-**
Hadar	**+**	**-**	**-**
Corvallis	**+**	**-**	**-**
Rissen	**+**	**-**	**-**
Paratyphi A	**+**	**+**	**-**
Bareilly	**+**	**-**	**-**
Newport	**+**	**-**	**-**
Agona	**+**	**-**	**-**
Tennessee	**+**	**-**	**-**
Typhimurium	**+**	**-**	**-**
Weltevreden	**+**	**-**	**-**
Enteritidis	**+**	**-**	**-**
Albany	**+**	**-**	**-**
Paratyphi B	**+**	**-**	**-**
Paratyphi C	**+**	**-**	**-**
Braenderup	**+**	**-**	**-**
Infantis	**+**	**-**	**-**
**Non-salmonella species**			
*Klebsiella pneumonia*	**-**	**-**	**-**
*Pseudomonas aeruginosa*	**-**	**-**	**-**
*Escherichia coli*	**-**	**-**	**-**
*Shigella flexnari*	**-**	**-**	**-**
*Vibrio cholerae*	**-**	**-**	**-**
*Acenitobacter baumannii*	**-**	**-**	**-**
*Aeromonas hydrophila*	**-**	**-**	**-**
*Neisseria meningitidis*	**-**	**-**	**-**
*Streptococcus spp*	**-**	**-**	**-**
*Staphylococcus epidermidis*	**-**	**-**	**-**
*Providencia spp*	**-**	**-**	**-**
*Enterococcous feacalis*	**-**	**-**	**-**
*Citrobacter freundii*	**-**	**-**	**-**
*Proteus mirabilis*	**-**	**-**	**-**
*Serratia marcerscens*	**-**	**-**	**-**

+ presence,

- absence

### Determination of the mPCR detection limit for spiked fecal samples

**Fig 7:**
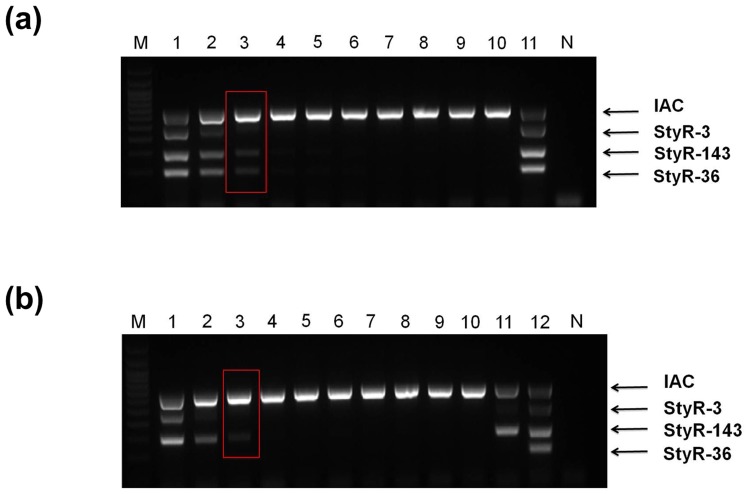
Analytical sensitivity of serially diluted overnight grown bacterial culture spiked in human feces prior to enrichment on 2% agarose gel electrophoresis. **a) *S*. Typhi.** Lane M: 100bp DNA ladder (Promega), Lane 1–9: represents DNA extracted from human feces spiked with different concentrations of *S*. Typhi before 4hrs of enrichment (1.5 X 10^8^ CFU/ml—1.5 X 10^0^ CFU/ml), Lane 10: DNA of unspiked human feces, lane 11: *S*. Typhi gDNA, Lane N: Negative control and **b) *S*. Paratyphi A.** Lane M: 100bp DNA ladder (Promega), Lane 1–9: represents DNA extracted from human feces spiked with different concentration of *S*. Paratyphi A before 4hrs of enrichment (1.5 X 10^8^ CFU/ml—1.5 X 10^0^ CFU/ml), lane 10: DNA of unspiked human feces, lane 11: *S*. Paratyphi A gDNA, lane 12: *S*. Typhi gDNA. Lane N: Negative control.

**Fig 8:**
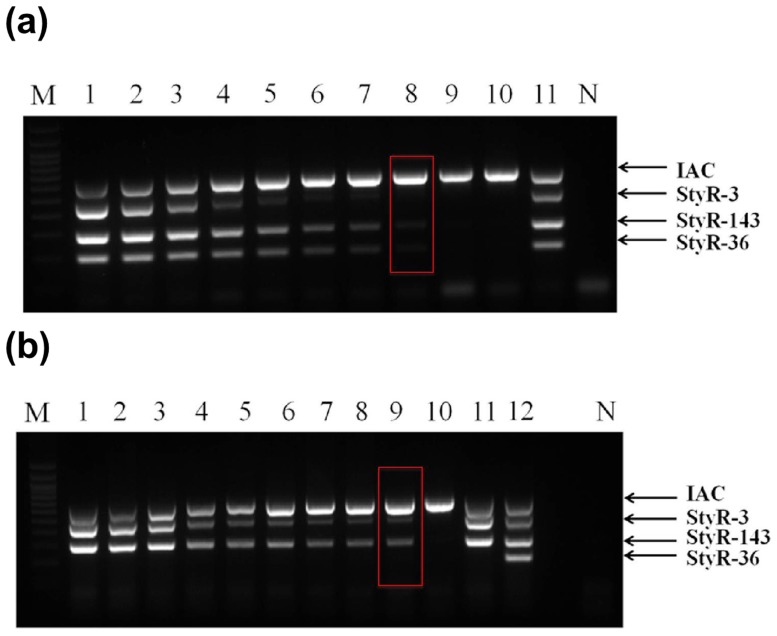
Analytical sensitivity of serially diluted overnight grown bacterial culture spiked in human feces after 4h of enrichment on 2% agarose gel electrophoresis. **a) *S*. Typhi.** Lane M: 100bp DNA ladder (Promega), Lane 1–9: represents DNA extracted from human feces spiked with different concentration of *S*. Typhi after 4hrs of enrichment (1.5 X 10^8^ CFU/ml—1.5 X 10^0^ CFU/ml), Lane 10: DNA of unspiked human feces, lane 11: *S*. Typhi gDNA, Lane N: Negative control and **b) *S*. Paratyphi A.** Lane M: 100bp DNA ladder (Promega), Lane 1–9: represents DNA extracted from human feces spiked with different concentrations of *S*. Paratyphi A after 4hrs of enrichment (1.5 X 10^8^ CFU/ml—1.5 X 10^0^ CFU/ml), lane 10: DNA of unspiked human feces, lane 11: *S*. Paratyphi A gDNA, lane 12: *S*. Typhi gDNA. Lane N: Negative control.

Stool spiking was performed to emulate clinical samples. Performance of the mPCR on the spiked samples is vital to evaluate the utility of mPCR for the direct detection of *S*. Typhi and *S*. Paratyphi A in fecal samples. The detection limit for both *S*. Typhi- and *S*. Paratyphi A-spiked feces was 1.5 x 10^6^ CFU/mL before enrichment (lane 3, Fig. 7a & b). However, with 4 h of enrichment in selenite cysteine broth, the sensitivity increased to 15 CFU/mL (lane 8, Fig. 8a) and 1.5 CFU/mL (lane 9, Fig. 8b) for *S*. Typhi and *S*. Paratyphi A, respectively. Notably, these detection limits are better than results obtained in other recent studies, which ranged from 1.0 x 10^2^ to 5.5 x 10^4^ CFU/mL [48,34,49]. The ability to identify *Salmonella* in fecal samples is advantageous because it allows for diagnosis of asymptomatic carriers. Indeed, 5% of typhoid patients become chronic carriers, shedding the organism in their feces after recovery [50]. Upon ingestion of the organisms, the likelihood and the severity of infection depend on the ingestion dose, the virulence of the *Salmonella* strain, and the status of host defense mechanisms. Usually, an infectious dose of 10^3^ and 10^5^ of non-typhoidal serovars and enteric serovars, respectively, are required to produce clinical infection in normal hosts [51]. Therefore, the sensitivity of the mPCR obtained conveys its potentiality of detecting salmonella in infected patients at the genomic DNA level.

Here, we have reported the efficacy of using novel sRNA genes (*StyR-3*, *StyR-36*, and *StyR-143)* as targets in mPCR to detect and differentiate salmonellosis. sRNAs represent superior candidates for molecular targeting assays, especially in PCR and reverse-transcription quantitative real-time PCR diagnostics (RT-qPCR). Indeed, we have demonstrated that mPCR is useful for specific microbiological detection of *S*. Typhi. Efficient diagnosis of *S*. Typhi is essential for successful treatment and disease surveillance programs including monitoring and transmission [49]. The increased infection rate of *S*. Paratyphi A in developing countries, especially among typhoid-vaccinated travelers, has led to more cases of enteric fever globally. Therefore, the sRNA-based mPCR assays shown here, offer excellent specificity and represent a way to improve control and surveillance strategies for these enteric pathogens.

## Conclusions

Collectively, our findings demonstrate that sRNA genes serve as excellent molecular biomarkers for the effective diagnosis of bacterial infections. In particular, our novel, sRNA-based mPCR assay may represent an alternate method for the surveillance of clinically important *Salmonella* strains. The results of the present study support the use of sRNA genes in other molecular-based diagnostic methods, such as nucleic acid sequence-based amplification (NASBA) [52]. Similarly, it can also be applied in other isothermal-based nucleic acid amplification strategies such as strand displacement amplification (SDA) and loop-mediated isothermal amplification [53]. Furthermore, sRNA candidates are amenable for use in real-time detection of live *Salmonella* species via RT-qPCR diagnostics [54].
